# 

**Published:** 1804-04-01

**Authors:** 


### TO CORRESPONDENTS.

Dr. LAMBE's Researches into the Properties of Spring Water, with Medical Cautions against the Use of Lead in the Construction of Pumps, Water Pipes, Cisterns, &c. will be noticed in our next Number.

Communications are received from Mr. Bartlett, &c.

Many of our Readers having requested a General Index of Diseases, Remedies, and Persons, for every *ten* or *twelve* volumes, we desire to acquaint them that such an Index is now preparing.

A CORRESPONDENT, to whom we acknowledge ourselves indebted for several valuable papers, complains of our having omitted and altered some expressions in his last communication; but we assure him that without such omissions and similar alterations, the Reply could not have appeared. It is the indispensable duty of the Editors to consider not merely what a wounded Author may feel, but the language in which his feelings ought to be expressed when transmitted for insertion in the Medical and Physical Journal, as the Nature of the Work cannot admit of its being made the Vehicle of Invective.

